# Educational differences in macro-level determinants of early exit from paid work: a multilevel analysis of 14 European countries

**DOI:** 10.1007/s10433-019-00538-6

**Published:** 2019-10-16

**Authors:** Sascha de Breij, Martijn Huisman, Dorly J. H. Deeg

**Affiliations:** 1 Department of Epidemiology and Biostatistics, Amsterdam Public Health Research Institute, Amsterdam UMC, Vrije Universiteit Amsterdam, De Boelelaan 1089A, 1081 HV Amsterdam, The Netherlands; 2 Department of Sociology, VU University Amsterdam, De Boelelaan 1081, 1081 HV Amsterdam, The Netherlands

**Keywords:** Early work exit, Institutional determinants, SHARE, European comparison, Educational differences

## Abstract

The aim of this study was to identify macro-level determinants of early work exit and investigate whether the effects of these determinants differ across educational groups. We used data from the Survey on Health, Ageing and Retirement in Europe (SHARE) (2011–2013) and the English Longitudinal Study of Ageing (ELSA) (2010/2011–2012/2013) as well as macro-level data and included 10,584 participants in 14 European countries. We used logistic multilevel analyses to examine educational differences in macro-level determinants of early work exit. Macro-level determinants were: minimum unemployment replacement rates, expenditure on active labour market policies (aimed to help the unemployed find work) and passive labour market policies (unemployment and early retirement benefits), employment protection legislation (costs involved in dismissing individuals), unemployment rates, statutory pension age and implicit tax on continued work. We found low-educated workers to be more at risk of early work exit than higher educated workers. In low-educated men, higher unemployment replacement rates, higher expenditure on passive labour market policies, stricter employment protection legislation and a higher implicit tax on continued work were associated with a higher risk of early work exit, whereas no macro-level factors were associated with early work exit in highly educated men. In women, a higher expenditure on passive labour market policies and a higher implicit tax on continued work were determinants of early work exit, regardless of educational level. To conclude, low-educated men seem to be especially responsive to the effects of pull factors that make early retirement financially more attractive.

## Introduction

In many industrialized countries, the population is ageing due to increasing life expectancy and decreasing fertility (Christensen et al. 2009). To reduce the economic burden that the ageing of society places on a welfare state, many countries are encouraging older workers to remain longer in the labour market by increasing the statutory retirement age, reducing generosity of benefits and/or restricting access to early retirement schemes. The actual retirement age, i.e. the age at which workers leave the labour market, however, continues to stay behind the statutory retirement age in most European countries (OECD 2014).

Studies show that there is variation in retirement timing across European countries (e.g. Ebbinghaus and Hofäcker 2013), suggesting that macro-level factors may play a role in retirement timing. To understand cross-country differences in retirement timing, a better understanding of the pension and labour market policies that affect early exit from the labour market is needed.

### Macro-level determinants

Decisions on early work exit are influenced by micro-level factors, with health being the most important determinant (Van Rijn et al. 2014), meso-level (workplace) factors (Scharn et al. 2018) and macro-level factors (Scharn et al. 2018). In this study, we focus on macro-level factors as determinants of early work exit. Macro-level factors may affect different routes of early exit from paid work, via public or private early retirement schemes, but also via social insurance programs (unemployment and disability). Determinants of retirement timing can be categorized into pull, push and maintain factors (Ebbinghaus and Hofäcker 2013; Hofäcker et al. 2016). Macro-level factors belonging to each of these groups were included in the present study.

Pull factors provide financially attractive opportunities for older workers to exit the labour market early, e.g. generous unemployment benefits and high implicit taxes on continued work. Previous studies have shown that in countries with more generous pension systems, early work exit is more prevalent (Blöndal and Scarpetta 1999; Duval 2003; Schils 2008). Most studies have examined replacement rates in this regard. A replacement rate is a measure of how generously a pension system provides income after work exit to replace earnings which were the main source of income prior to work exit (Korpi and Palme 2007b). A high unemployment replacement rate acts as a pull factor by providing a financially attractive alternative to continuing work. Higher implicit taxes on continued work also act as pull factors by making early retirement financially more attractive than continuing work (Blöndal and Scarpetta 1999; De Preter et al. 2013; Duval 2003). There is an implicit tax on continued work when the change in pension wealth from working one additional year is less than the value of contributions paid to the pension (Duval 2003). The implicit tax on continued work also captures some of the effects of eligibility ages and generosity of benefits. The higher the replacement rate, the higher is the implicit tax on continued work and the higher the minimum pensionable age, the lower is the implicit tax on continued work before this age (Duval 2003). In addition, expenditure on passive labour market policies (PLMP) (De Preter et al. 2013) may also be associated with early work exit. A high expenditure on PLMP, consisting of expenditure on unemployment benefits and early retirement schemes, may make early work exit more attractive, acting as a pull factor.

Maintain factors, e.g. training older workers, job creation or start-up incentives, promote older workers’ employability. A high expenditure on active labour market policies (ALMP) which comprise policies aimed at improving access to the labour market for the unemployed and disabled, may decrease the rate of early work exit (Ebbinghaus and Hofäcker 2013; Martin 2015; Martin and Grubb 2001) and can therefore be seen as a maintain factor.

Push factors make it more difficult or less attractive to stay in the labour market and therefore ‘push’ workers out of the work force. There is evidence that strictness of employment protection legislation (EPL) is related to early work exit (Bellmann and Florian 2007; Dorn and Sousa-Poza 2007; Ebbinghaus and Radl 2015). A strict EPL may make firms less likely to hire older workers because of expensive future dismissals. This could in turn lead to forcing older workers who can no longer work for their present employer or who were already unemployed into unemployment. Firms ‘pushing’ their older employees into early retirement instead of making them redundant (which is harder under strict EPL) may be another explanation for this positive association (Ebbinghaus and Radl 2015). In this way, a strict EPL can be seen as a push factor. It could also be argued, however, that it acts as a maintain factor, with older workers who are still employed and can still carry out their jobs being better protected and are therefore less at risk of early work exit. Some studies did not find any effect of EPL on early work exit (De Preter et al. 2013; Fischer and Sousa-Poza 2006). Another push factor is a high unemployment rate. Higher unemployment rates have been shown to be associated with early work exit (Gorodnichenko and Song 2013), most likely through discouragement, as unemployment rates reflect the chances of finding a new job (Blöndal and Scarpetta 1999; Fischer and Sousa-Poza 2006). A high statutory pension age may also have a ‘push’ effect. When workers have to work up to higher ages, the risk of early exit due to health reasons may increase (Ebbinghaus and Radl 2015).

### Education

Reasons why workers exit the work force early vary by educational level. On the one hand, workers with a low education usually exit the work force earlier than higher educated workers (Denaeghel et al. 2011; Jensen et al. 2012; Visser et al. 2016), which is mostly through disability and unemployment pathways. On the other hand, lower educated workers are more inclined to work longer because of financial necessity (Radl 2013). With more generous pension systems, these financial reasons may become less important, and may therefore increase the probability of early work exit among lower educated workers.

So far, little research has been done on educational differences in macro-level determinants of early work exit. Pension policies may not have the same effect on workers with different educational levels (Buchholz et al. 2013; Schils 2008). Because lower educated workers more often leave the work force through unemployment than higher educated workers (Schuring et al. 2013; Van Zon et al. 2017), we hypothesize that a higher expenditure on PLMP is a stronger predictor of early work exit in lower educated workers, who make more use of the benefits that PLMP offer. For the same reason, we also expect expenditure on ALMP, aimed at improving access to the labour market for the disabled and unemployed, to be a stronger predictor in the lower educated group, but in the opposite direction, with lower expenditure being associated with a higher risk of early work exit. In addition, higher unemployment replacement rates and higher implicit taxes on continued work may also be more strongly associated with early exit in the lower educated, given that this reduces their financial necessity to continue working.

Strictness of EPL may also be more important for lower educated workers, who are more often seen by managers as having trouble with adaptability to changes than higher educated workers (Henkens 2010). With a less strict EPL, these workers can be laid off more easily, with less financial consequences for the employer. It could also be argued, however, that a stricter EPL leads to firms pushing these lower educated workers into involuntary retirement. We hypothesize that a strict EPL acts as a maintain factor in higher educated workers, but as a push factor in lower educated workers.

And finally, a higher statutory pension age may also be more strongly associated with early work exit in lower educated workers, because lower educated people have poorer health compared to the higher educated (see e.g. Mackenbach et al. 2008), and may therefore not be able to work up to higher ages due to health problems.

### Sex differences

In many countries, the statutory pension age differed between men and women in the last decades, but differences are generally being phased out (OECD 2011). Previous studies also show that there are sex differences in retirement intentions and behaviours (Albertsen et al. 2007; Dahl et al. 2003) and in the effect of macroeconomic factors on retirement decisions (Axelrad and McNamara 2017; Shuey and O’Rand 2004). Older female workers, for example, may have to work longer to be entitled to the same benefits as men, because they are more likely to have had interruptions in their working careers (Musumeci and Solera 2013). Therefore, we will consider possible sex differences when examining determinants of early work exit.

### Present study

So far educational differences have been largely overlooked in research on determinants of early work exit. We contribute to the existing research by combining micro-level and macro-level data and examining cross-level interactions to identify educational differences in the effect of macro-level factors on early work exit. We examined whether (1) there are educational differences in early work exit and (2) which macro-level factors are determinants of early work exit. Our main research question was (3) whether there are educational differences in the effect of these macro-level factors on early work exit.

## Methods

### Sample

**Fig. 1:**
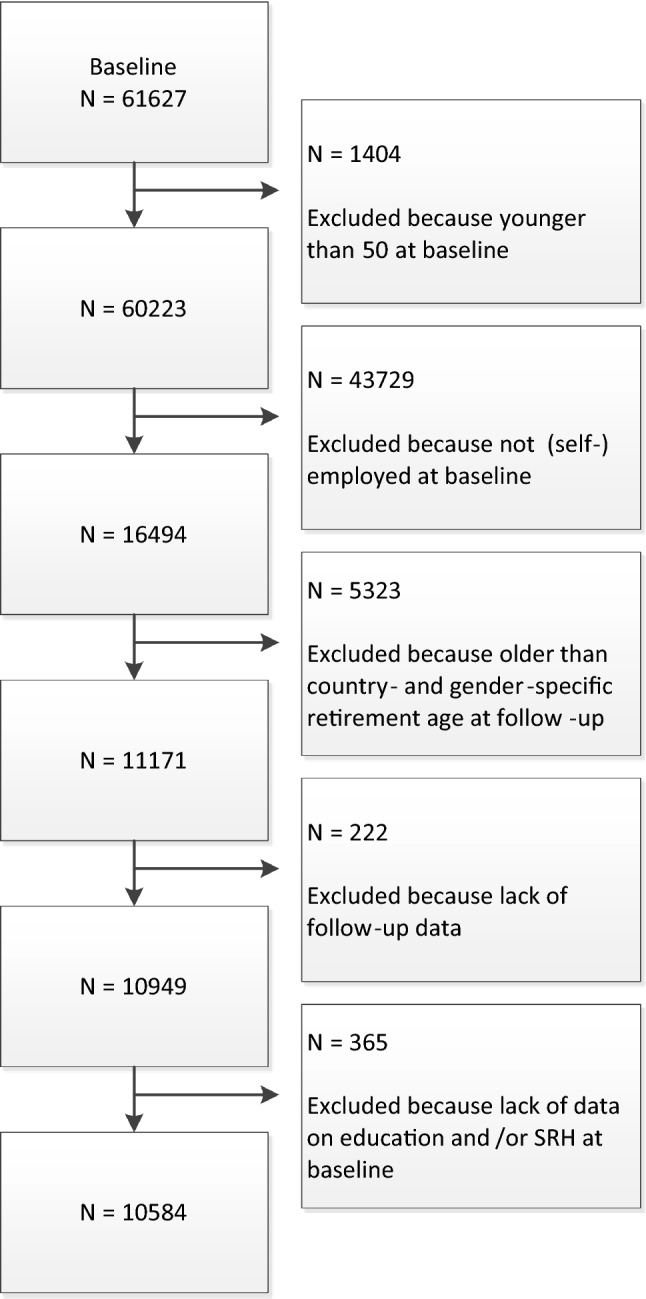
Selection of the sample

We used micro-level data from two harmonized longitudinal studies on ageing: the Survey of Health, Ageing and Retirement in Europe (SHARE) and the English Longitudinal Study of Ageing (ELSA). We used wave 4 (2011) and 5 (2013) of SHARE and included all countries that participated in both waves: Austria, Belgium, the Czech Republic, Denmark, Estonia, France, Germany, Italy, the Netherlands, Slovenia, Spain, Sweden, Switzerland. For the English data, we used wave 5 (2010/2011) and 6 (2012/2013) of ELSA and pooled them with the SHARE waves. We ended up with 14 countries in our sample. In each country, nationally representative samples of households with individuals aged 50 and older were drawn. Details on the SHARE and ELSA studies have been described elsewhere (Börsch-Supan et al. 2013; Steptoe et al. 2013). Starting at baseline with a total sample of 61,627 respondents, we included respondents who were 50 years or older and reported having paid work (*N* = 16,494). Respondents were excluded if they were older than the country- and sex-specific statutory retirement age at follow-up (*N* = 5323), lacked data at follow-up (*N* = 222), or had missing data on education (*N* = 274) or self-rated health (SRH) (*N* = 91) at baseline. Our final sample included 10,584 respondents (Fig. 1).

### Measures

#### Outcome

##### Early work exit

Respondents were asked to choose the best description of their current situation: retired, employed, self-employed, unemployed, permanently sick or disabled, looking after home or family or other. We classified respondents as having paid work if they answered ‘employed’ or ‘self-employed’, and as having exited the work force early if they answered otherwise. Our outcome was early work exit versus still having paid work at two-year follow-up.

#### Micro-level explanatory variable

##### Education

To measure educational level, the International Standard Classification of Education 2011 (ISCED 2011) was used, which provides a framework to classify diverse educational systems in such a way that they can be compared across countries. Scores, ranging from 0 (no education) to 6 (second stage of tertiary education), were categorized into three groups: low (up to lower secondary education), intermediate (upper secondary education or post-secondary non-tertiary education) and high (short cycle tertiary and higher) education.

#### Macro-level explanatory variables

For the macro-level factors, we derived data from the OECD databases and the Social Insurance Entitlement Dataset (SIED), a continuation of the Social Citizenship Indicator Program (SCIP) (Korpi and Palme 2007a). Macro-level factors were all measured in 2011 (at baseline) except for the unemployment replacement rate and implicit tax on continued work (2010) due to availability of the data.

##### Unemployment replacement rate

We included minimum unemployment replacement rates (RR). RR refer to what extent the insurance program replaces the average production worker’s wage (APWW) and is calculated as the ratio of the minimum replacement level divided by the APWW (Korpi and Palme 2007a). The minimum replacement level refers to the floor below which a worker cannot fall. These benefits can be income tested. All calculations are based on a single standard worker in the manufacturing (metal-) industry with certain characteristics, to make cross-country comparison possible. The unemployment RR are based on a single standard worker who:

- is 30 years of age;
- has worked for 10 years;
- worked for 5 years at the present employer;
- was not unemployed during the last 2 years;
- is assumed not to be living with their family of origin.

##### Expenditure on labour market policies

Expenditure on active labour market policies (ALMP) (as a percentage of GDP) includes all social expenditure which is aimed at helping the unemployed to return to the labour market (e.g. training, employment incentives, direct job creation, supported employment and rehabilitation, start-up incentives). Expenditure on passive labour market policies (PLMP) (as a percentage of GDP) refers to income replacements, namely unemployment benefits and early retirement benefits (OECD 2013a). The disadvantage of using expenditure measures is that a higher expenditure does not necessarily imply a greater effort, it could also indicate that there is a greater need in that specific country. In this study, all models including expenditure on LMP will be adjusted for unemployment rate as a proxy for need.

##### Employment protection legislation

The OECD employment protection index is compiled from sub-components quantifying, for employers, the costs and procedures involved in dismissing individuals—or groups of employees (OECD 2013a). We used version 3 of the index for individual and collective dismissals of workers with regular contracts. The index ranges from 0 to 6, with higher scores representing stricter regulation.

##### Unemployment rate

Unemployment rate is the number of unemployed people as a percentage of the labour force, i.e. the unemployed plus those in paid or self-employment (OECD 2018).

##### Implicit tax on continued work

We included the implicit taxes on continued work in the regular old-age pension system and in the early retirement route. The implicit tax rate on continued work (in percentages) is defined as the loss in pension wealth accrual from working for an additional year, where net pension wealth is defined as the present value of the future stream of pension payments to which a person is entitled. If the cost in terms of foregone pensions and contributions paid is exactly offset by an increase in future pension benefits, the pension system is said to be ‘actuarially neutral’, but if this cost is not offset, there is an implicit tax on continued work (Duval 2003). The implicit tax on continuing work for five more years was computed for a ‘standard’ single worker with average earnings at age 60 for the regular old age pension route and as an average for 55- and 60-year-old workers for the early retirement route (OECD 2013b). In countries where unemployment-related benefits can be used to bridge the time until people are entitled to an old-age pension, implicit taxes on continued work are also computed for these benefits. If unemployment-related schemes cannot be used in such a way, other schemes are used, e.g. disability schemes. If there are no social programs that facilitate exit from the labour market before the statutory pension age, then the old-age pension pathway is used (Duval 2003).

##### Statutory pension age

The gender-specific statutory pension age (SPA) of each country was included (OECD 2013b).

All macro-level factors were centred around their grand mean for a better interpretation of the cross-level interactions.

#### Control variables

We analysed men and women separately. Additionally, we controlled for age and for self-rated health (SRH), since health is the most important micro-level factor affecting early work exit. SRH was measured with one question: “Would you say your health is ….”. The answer categories were: “excellent” (1), “very good” (2), “good” (3), “fair” (4), or “poor” (5). This question is widely used as a measure of health (Jylhä 2009). Age was categorized into three categories, based on the odds of early work exit in our sample: 50–56, 57–62, 63–64 years. Furthermore, we controlled for the macro-level factor GDP per capita.

### Statistical analysis

The analyses were conducted in Stata version 14. We used multilevel models, because in our data individuals (level 1) were nested in countries (level 2). As the outcome was binary, we used multilevel logistic regression models. All analyses were stratified by sex. First, an intercept-only model with a random intercept for country level was built (model 1). Likelihood ratio tests were used to test whether the model with a random intercept was better than a model without a random intercept. Random slopes were added for education and age, to adjust for country differences in the effect of these variables. Because measures such as the intraclass correlation coefficient (ICC) are very difficult to interpret for a dichotomous variable, we used the median odds ratio (MOR) as a measure of heterogeneity (Merlo et al. 2006), to explore between-country differences in early work exit. The MOR is defined as the median value of the odds ratio between the country at highest risk and the country at lowest risk when randomly picking out two countries. The MOR can be interpreted as the median increased odds of retiring early if an individual moves to another country with higher risk. Therefore, the higher the MOR, the greater is the between-country variance. If there would be no variation between countries, the MOR would be 1. In model 2a, the crude effect of educational level on early work exit was modelled, and in model 2b, this effect was adjusted for the confounders, to answer our first research question: ‘Are there educational differences in early work exit?’.

Next, we built separate models for each macro-level factor and adjusted for the confounders and education (model 3) to answer research question 2: ‘Which macro-level factors are determinants of early work exit?’.

Finally, we added cross-level interactions between education and the macro-level factors to answer research question 3: ‘Are there educational differences in the effect of macro-level factors on early work exit?’.

## Results

Table 1 shows the micro-level characteristics of the sample. The percentage of men who retired early was smallest in Denmark and largest in Spain. For women, percentage of early work exit was smallest in Estonia and largest in the Czech Republic. The descriptive statistics show that high percentages of early work exit are not more apparent in countries with a higher baseline age or a lower statutory pension age. In Sweden for example, the age at baseline is highest (59.6 years), and the statutory pension age is average (65 years), but the percentage early work exit for men is among the lowest (10.5%). Another example is Slovenia, which has the second highest percentage of early work exit (26.3% for men and 22.2% for women), but has the lowest baseline age (54.7 years for men and 53.6 years for women) and the second lowest statutory pension age (63 years for men and 61 for women). Cross-country differences in the educational distribution were also evident.

**Table 1:** Micro-level characteristics per country

	Early work exit at follow-up %	Statutory pension age	Age at baseline *M* (SD)	Low education (%)	Intermediate education (%)	High education (%)	SRH *M* (SD)	*N*
*Men*								
Austria	19.8	65	55.2 (3.5)	6.2	58.0	35.8	2.5 (0.9)	419
Belgium	16.5	65	55.3 (3.5)	26.3	28.6	45.1	2.6 (0.9)	479
Czech Republic	16.8	62	55.6 (3.0)	43.4	36.7	19.9	2.9 (1.0)	357
Denmark	9.6	65	55.8 (3.7)	7.7	46.0	46.3	2.1 (0.9)	376
England	18.3	65	58.4 (3.0)	20.5	51.6	27.9	2.4 (1.0)	927
Estonia	14.7	63	55.5 (3.3)	13.7	62.2	24.2	3.4 (0.9)	513
France	19.6	65	55.0 (3.2)	17.7	52.1	30.3	2.8 (1.0)	459
Germany	17.0	65	58.8 (2.8)	2.0	47.0	51.0	3.0 (0.9)	100
Italy	23.9	66	56.4 (3.5)	46.6	41.0	12.4	2.6 (0.9)	234
Netherlands	18.8	65	56.8 (3.6)	27.5	32.6	39.9	2.6 (0.9)	298
Slovenia	26.3	63	54.7 (3.0)	10.9	64.6	24.6	2.8 (1.0)	175
Spain	27.9	65	56.6 (3.7)	62.2	18.4	19.4	2.7 (0.9)	294
Sweden	10.5	65	59.6 (2.3)	31.5	37.0	31.5	2.4 (1.0)	143
Switzerland	10.6	65	56.5 (3.8)	6.3	69.9	23.8	2.4 (0.9)	509
Total	17.4	*M *= 64.6 (SD = 1.0)	56.4 (3.6)	21.7	48.1	30.2	2.6 (1.0)	5283
*Women*								
Austria	19.2	60	53.8 (2.6)	19.5	43.8	36.7	2.4 (0.9)	349
Belgium	13.3	65	54.7 (3.3)	21.8	33.0	45.2	2.7 (0.9)	518
Czech Republic	29.3	61	54.5 (2.6)	30.9	53.5	15.6	2.9 (0.9)	417
Denmark	16.2	65	55.5 (3.6)	7.8	27.9	64.3	2.2 (1.0)	387
England	15.6	61	55.8 (2.4)	23.8	54.0	22.2	2.4 (1.0)	807
Estonia	7.4	61	54.3 (2.8)	8.1	56.3	35.6	3.3 (0.9)	618
France	16.8	65	54.9 (3.2)	28.3	42.1	29.6	2.8 (1.0)	523
Germany	19.5	65	58.1 (2.6)	6.8	52.0	41.2	2.9 (0.9)	148
Italy	12.3	62	54.7 (2.8)	36.3	46.2	17.5	2.8 (1.0)	171
Netherlands	12.9	65	55.9 (3.5)	29.5	31.6	38.9	2.6 (1.0)	288
Slovenia	22.2	61	53.6 (2.3)	10.8	50.0	39.2	2.8 (0.9)	158
Spain	14.8	65	55.5 (3.7)	53.8	24.3	21.9	2.8 (0.9)	210
Sweden	17.6	65	59.1 (2.8)	22.3	35.1	42.6	2.4 (1.1)	188
Switzerland	10.2	64	55.7 (3.5)	13.7	65.9	20.4	2.4 (0.9)	519
Total	15.5	*M *= 63.0 (SD = 2.0)	55.2 (3.2)	20.9	46.2	32.9	2.7 (1.0)	5301

*SRH* Self-rated health

Table 2 shows the macro-level characteristics per country. With regard to unemployment, England (0.12) had the least generous benefits whereas Slovenia (0.79) had the most generous benefits. There were also large cross-country differences in ALMP expenditure, ranging from 0.22 (Estonia) to 1.6 (Sweden), and in PLMP expenditure, ranging from 0.31 (England) to 2.82 (Spain). Belgium (3.13) had the strictest and England (1.76) the least strict EPL. Unemployment rates were highest in Spain (21.4) and lowest in Switzerland (4.4). The implicit tax on continued work in old age pension systems were highest in Slovenia (78.77% of average earnings) and lowest in Denmark (− 0.15% of average earnings) and in early retirement they were highest in Spain (64.38% of average earnings) and lowest in the Netherlands (2.81% of average earnings).

**Table 2:** Macro-level characteristics per country

	GDP per capita (/1000)	Unemployment RR	ALMP expenditure	PLMP expenditure	EPL	Unemployment rate	Implicit tax old age pension	Implicit tax early retirement
Austria	44.5	0.45	0.73	1.25	2.44	4.6	5.92	58.59
Belgium	41.5	0.76	0.84	2.04	3.13	7.1	32.71	28.50
Czech Republic	28.8	0.60	0.26	0.27	2.75	6.7	17.39	17.96
Denmark	44.4	0.58	2.02	1.60	2.32	7.6	− 0.15	3.92
England	36.6	0.12	0.23	0.31	1.76	8.0	13.85	22.14
Estonia	24.5	0.47	0.22	0.48	2.07	12.3	23.53	12.53
France	37.5	0.69	0.93	1.80	2.82	8.8	9.93	32.73
Germany	42.7	0.62	0.77	0.99	2.84	5.8	15.45	13.59
Italy	39.9	0.69	0.41	1.24	3.03	8.4	15.56	4.24
Netherlands	46.1	0.66	1.03	1.36	2.88	5.0	1.64	2.81
Slovenia	28.8	0.79	0.35	0.91	2.67	8.2	78.77	60.33
Spain	32.1	0.63	0.87	2.82	2.56	21.4	26.50	64.38
Sweden	43.8	0.54	1.16	0.60	2.52	7.8	25.13	18.57
Switzerland	56.2	0.71	0.56	0.51	2.10	4.4	18.84	17.47

*RR* Replacement rate, *ALMP* active labour market policies, *PLMP* passive labour market policies, *EPL* employment protection legislation

### Cross-country differences in early work exit

The intercept-only (null) model showed statistically significant cross-country differences in the odds of early work exit (Table 3, model 1). The MOR of 1.38 and 1.40 for men and women, respectively, means that a person from a country with a high risk of early work exit has 1.38 (men) or 1.40 (women) times the odds of exiting the work force early compared to a person from a country with a low risk of early work exit.

**Table 3:** Multilevel models with associations between education and macro-level factors and early work exit

	Men OR (SE)	Women OR (SE)
Model 1: Null model		
Between-country variance (*σ*_u0_^2^)	.115	.136
MOR	1.382	1.402
Model 2a: Education (crude)^a^		
Low education	1.914 (.262)**	1.679 (.225)**
Intermediate education	1.253 (.129)*	1.244 (.129)*
Model 2b: Education (adjusted)^b^		
Low education	1.980 (.317)**	1.620 (.257)**
Intermediate education	1.371 (.158)**	1.285 (.149)*
Model 3: Macro-level factors^c^		
Unemployment RR	6.654 (4.692)**	1.334 (1.095)
ALMP expenditure^d^	.927 (.345)	1.282 (.435)
PLMP expenditure^d^	1.994 (.327)**	1.546 (.209)*
EPL	2.036 (.577)*	1.268 (.437)
Unemployment rate	.993 (.039)	.947 (.036)
SPA	1.072 (.181)	.876 (.063)
Implicit tax old age pension	1.011 (.008)	1.004 (.008)
Implicit tax early retirement	1.015 (.006)*	1.013 (.006)*

*MOR* Median odds ratio, *RR* replacement rate, *ALMP* active labour market policies, *PLMP* passive labour market policies, *EPL* employment protection legislation, *SPA* statutory pension age

*p value ≤ 0.05; **p value ≤ 0.01

^a^Including a random intercept and a random slope for education

^b^Adjusted for age, SRH, and GDP per capita and including a random slope for education and age

^c^Adjusted for education, age, SRH and GDP per capita and including a random slope for education and age

^d^Adjusted for education, age, SRH, GDP per capita and unemployment rate and including a random slope for education and age

### Educational differences in early work exit

Men (OR = 1.980) and women (OR = 1.620) with a low education had higher odds of early work exit than their higher educated peers (Table 3, models 2). ORs for intermediate education were smaller than those for low education, but also suggested higher odds of early work exit in the intermediate groups compared to the highest educated groups. By including an interaction term between sex and education, we examined whether the sex differences in ORs were statistically significant. There was no statistically significant sex difference in the association between education and early work exit.

### Macro-level determinants of early work exit

There were four macro-level factors that were significantly associated with early work exit (Table 3, model 3) and we found statistically significant sex differences in some of these determinants. A higher unemployment RR and a stricter EPL were associated with a higher risk of early work exit, in men only. A higher expenditure on PLMP and a higher implicit tax in the early retirement route were associated with early work exit in both men and women.

### Educational differences in macro-level determinants of early work exit

Interaction effects between education and the macro-level factors are shown in Table 4. These interactions show whether there are educational differences in macro-level determinants of early work exit, i.e. whether the associations between the macro-level factors and early work exit differ across educational groups.

**Table 4:** Cross-level interactions between education and macro-level factors (adjusted for age, SRH and GDP per capita, and including a random slope for education and age)

	Macro-level factor		Macro-level factor * education	
	Men OR (SE)	Women OR (SE)	Men OR (SE)	Women OR (SE)
Unemployment RR * low education	1.045 (.841)	.523 (.517)	7.824 (4.039)**	2.698 (2.091)
Unemployment RR * intermediate education			3.583 (1.549)**	2.479 (1.378)
ALMP expenditure * low education	.818 (.358)	1.215 (.486)	1.056 (.387)	1.042 (.357)
ALMP expenditure * intermediate education			1.192 (.291)	1.074 (.244)
PLMP expenditure * low education	1.664 (.379)*	1.495 (.402)	1.159 (.227)	1.037 (.220)
PLMP expenditure * intermediate education			1.242 (.196)	1.034 (.175)
EPL * low education	1.214 (.444)	1.017 (.450)	1.951 (.623)*	1.265 (.472)
EPL * intermediate education			1.413 (.334)	1.252 (.333)
Unemployment rate * low education	.965 (.049)	.924 (.048)	1.027 (.037)	1.032 (.044)
Unemployment rate * intermediate education			1.046 (.033)	1.012 (.040)
SPA * low education	1.341 (.274)	.952 (.087)	.762 (.106)#	.892 (.069)
SPA * intermediate education		.859 (.103)	.942 (.053)	
Implicit tax old age pension * low education	.996 (.010)	.997 (.010)	1.022 (.010)*	1.002 (.010)
Implicit tax old age pension * intermediate education		1.012 (.007)#	1.011 (.007)	
Implicit tax early retirement * low education	1.007 (.008)	1.009 (.008)	1.005 (.008)	1.005 (.008)
Implicit tax early retirement * intermediate education		1.010 (.006)#	1.004 (.006)	

*RR* Replacement rate, *ALMP* active labour market policies, *PLMP* passive labour market policies, *EPL* employment protection legislation, *SPA* statutory pension age

#*p* value ≤ 0.10; *p value ≤ 0.05; **p value ≤ 0.01

The OR for macro-level factor (first two columns) represents the effect of the macro-level factor on early work exit for the high education group. ORs for the low and intermediate education groups can be calculated by multiplying the OR for macro-level factor by the OR for the interaction macro-level factor * education (next two columns). In men, the effect of unemployment RR on early work exit differed by educational level. The effect was much stronger in low (OR = 1.045 * 7.824 = 8.176, *p* = .011) and intermediately (OR = 1.045 * 3.583 = 3.744, *p* = .087) educated men, compared to men with a high educational level (OR = 1.045, *p* = .956). In low-educated men, a higher unemployment RR was statistically significantly associated with higher odds of early work exit, whereas in higher educated men unemployment RR did not have a significant effect on timing of work exit.

In men, stricter EPL was associated with higher odds of early work exit in the low-educated workers (OR = 2.369, *p* = .007), and to a lesser extent in the intermediately educated workers (OR = 1.716, *p* = .078), but not in those with a high education (OR = 1.214, *p* = .596).

We also found an interaction between SPA and education in men, but the association between SPA and early work exit was not statistically significant in any of the educational groups (low: OR = 1.022, *p* = .901; intermediate: OR = .897, *p* = .158; high: OR = 1.151, *p* = .428).

In men, the association between implicit tax on continued work and early work exit also differed statistically significantly across educational groups in both the regular old age pension route (low: OR = 1.019, *p* = .056; intermediate: OR = 1.008, *p* = .309; high: OR = .996. *p* = .706) and early retirement route (low: OR = 1.013, *p* = .048; intermediate: OR = 1.018, *p* = .005; high: OR = 1.007, *p* = .354), with stronger associations in the lower educated.

### Sensitivity analyses

Because we included only 14 countries, we performed sensitivity analyses to check the robustness of our findings. To make sure our results were not affected by outliers, we additionally conducted the analyses with dichotomized macro-level factors. These analyses yielded similar results compared to the results obtained with the continuous macro-level factors (results available upon request).

## Discussion

So far, research on macro-level determinants of early work exit has neglected possible educational differences. Our aim was to examine (1) whether there are educational differences in early work exit, (2) which macro-level factors are determinants of early work exit, and (3) whether there are educational differences in the effect of these macro-level factors on early work exit.

Consistent with earlier findings (e.g. Ebbinghaus and Hofäcker 2013), we found cross-country differences in early work exit. Results from our multilevel analyses further show that those with a low education generally have a higher risk of exiting the work force early than those with a high education. This finding is consistent with the literature (Denaeghel et al. 2011; Jensen et al. 2012; Visser et al. 2016).

Previous studies have shown associations between early work exit and generosity of benefits (Blöndal and Scarpetta 1999; Duval 2003; Schils 2008), expenditure on active labour market policies (ALMP) and passive labour market policies (PLMP) (De Preter et al. 2013; Martin and Grubb 2001), strictness of employment protection legislation (EPL) (Bellmann and Florian 2007; Dorn and Sousa-Poza 2007; Ebbinghaus and Radl 2015), and unemployment rates (Blöndal and Scarpetta 1999; Fischer and Sousa-Poza 2006). We extended these findings by showing that a higher expenditure on PLMP and a higher implicit tax on continued work in early retirement schemes were associated with a higher risk of early work exit in both men and women. In men, a higher unemployment replacement rate and a stricter EPL were also associated with a higher risk of early work exit. We did not find an association between unemployment rates and early work exit. Our selection of a sample of employed persons could explain this lack of effect: those already employed may not be as affected by unemployment rates compared to the unemployed trying to find a job.

Addressing our third research question, we found that the macro-level pull factors, which make early retirement financially more attractive, seem to have an effect only on the lower educated workers. The educational differences in the effects of these pull factors, i.e. unemployment replacement rates, expenditure on PLMP, and implicit taxes on continued work, support our hypothesis that low-educated workers’ financial reasons to continue working become more important when facing less generous benefits. We hypothesized that strictness of EPL would be a push factor for the lower educated and a maintain factor for the higher educated workers. Indeed, we found evidence to support the first part of this hypothesis. So although a strict EPL may be seen as a protective measure, this protectiveness is not applicable to workers with a low educational level. These lower educated workers, who generally have more physically demanding jobs and who are seen as less adaptable by their employers (Henkens 2010), seem to be ‘pushed’ out of the labour market through alternative routes instead of being made redundant when facing strict EPL (Ebbinghaus and Radl 2015). We did not find evidence that a strict EPL was a maintain factor for highly educated workers.

We did not find an association between ALMP expenditure and early work exit, regardless of educational level. This may be due to the characteristics of our sample: we selected respondents who had paid work at baseline and follow-up was only 2 years later. The expenditure on these policies, aimed at getting unemployed and disabled people back into the work force, may therefore be less applicable to our specific sample of older workers. It could also be argued that this measure of ALMP spending may not be ideal for this specific group of older workers, since it reflects spending on the entire population and may be biased towards other age groups (Hofäcker and Unt 2013).

We also did not find an association between the statutory pension age and early work exit. This does not necessarily mean that SPA does not affect early work exit. This lack of effect may be due to the small variation in SPA, especially in men.

It is important to keep in mind that these macro-level factors not only have a negative effect, i.e. increase the risk of early exit from work, but also contribute positively to older workers and retirees. Higher replacement rates and a higher social expenditure are also associated with better population health (Bergqvist et al. 2013). Some workers can no longer work due to health problems and these policies give them the opportunity to exit the work force early. Work place interventions and health promotion are important in enabling this group of workers to continue working (De Boer et al. 2004; Oakman et al. 2018).

Strengths of this study are the combination of micro-level and macro-level data and the inclusion of cross-level interactions to identify educational differences. Our study also has some limitations. We could include only 14 countries in our multilevel analyses, which resulted in low statistical power. Because of this power issue, we could not fit full models to examine the effects of multiple macro-level determinants on early work exit. Future research including more countries, should therefore replicate our findings. In addition, while in the current study we only examined macro-level factors, further research should also investigate how these macro-level factors interact with factors on the meso-level. Also, the OECD EPL index we used as a measure of strictness of EPL has its disadvantages. Like any synthetic index it does not take into account the extent of the enforcement of laws (Myant 2016). Therefore, any effects of EPL should be interpreted with caution. We operationalized early work exit as having exited the work force before reaching the statutory pension age at 2 year follow-up. A limitation of this operationalization is that someone who does not work at follow-up may continue working later on and someone who is still working at follow-up could still exit work early, especially given the relatively low age at follow-up. Ideally one would want to follow each respondent up to the statutory pension age, but unfortunately this was not possible, because in SHARE not all countries participated in each wave.

To conclude, our study identified several macro-level factors that are associated with early work exit. It is clear, however, that educational differences as well as sex differences should be taken into account when examining the effects of these factors. Low-educated men seem to be especially responsive to the effects of pull factors that make early retirement financially more attractive.
